# Epidemiological evidence in law: a comment on Supreme Court Decision 2011Da22092, South Korea

**DOI:** 10.4178/epih/e2015025

**Published:** 2015-05-31

**Authors:** Alex Broadbent

**Affiliations:** Department of Philosophy, University of Johannesburg, Johannesburg, South Africa

**Keywords:** Law, Probability of causation, Smoking, Tobacco, Lung neoplasms

## Abstract

This paper offers a commentary on three aspects of the Supreme Court’s recent decision (2011Da22092). First, contrary to the Court’s finding, this paper argues that epidemiological evidence can be used to estimate the probability that a given risk factor caused a disease in an individual plaintiff. Second, the distinction between specific and non-specific diseases, upon which the Court relies, is shown to be without scientific basis. Third, this commentary points out that the Court’s finding concerning defect of expression effectively enables tobacco companies to profit from the efforts of epidemiologists and others involved in public health to raise awareness of the dangers of smoking.

## INTRODUCTION

The Korean Supreme Court recently announced a number of findings concerning the relevance of epidemiological evidence for proving individual causation in connection with smoking and lung cancer. This commentary identifies three elements of the decision that are of potential interest to epidemiologists and others involved in public health, both within and outside Korea:

1. Whether it is possible to prove causation in an individual plaintiff using epidemiological evidence;
2. Whether there is a real scientific or factual difference between “specific” and “non-specific” diseases, as identified by the Court;
3. Whether the judgement was correct in holding that tobacco companies had adequately warned smokers of the dangers of smoking.

The first issue is a scientific question, but is difficult to resolve because it does not fall within the domain of any single science. Thus, it cannot be settled merely by appealing to an expert witness. The analysis in my comment below therefore sets out a series of logical inferences from epidemiological evidence.

The second issue is a scientific question belonging to the realms of epidemiology and medicine, and can be settled by appeal to those disciplines. The comments below present the main scientific issues and the standard resolution thereof.

The third issue pertains to legal and social policy. The Court held that there was no defect in expression in relation to the dangers of smoking, because the dangers of smoking are already well-known. The fact they are well-known is due in part to the efforts of epidemiologists, and this judgement relieves tobacco companies of the need to compensate the epidemiological profession or the public health establishment for the associated costs. Epidemiologists both within Korea and abroad may well be dismayed to learn that their efforts to raise awareness of the health dangers of smoking have relieved tobacco companies of the costs of doing so.

## PROVING CAUSATION USING EPIDEMIOLOGICAL EVIDENCE

The Court’s main stance on this issue is summarized in 4(A) as follows:

…*in contrast with “specific diseases,” which have specific causes and clearly corresponding cause and effect, “non-specific diseases” have complicated and numerous causes and mechanisms and result from combinations of innate factors such as genes and bodily constitution, and acquired factors such as drinking, smoking, age, eating habits, occupational or environmental factors, etc. Even if an epidemiological correlation between a specific risk factor and the non-specific disease is acknowledged, the correlation simply means that exposure to the risk factor means the existence or increase of the risk of developing the disease and does not necessarily lead to the conclusion that the risk factor is the cause of the disease, as long as there is the possibility that the individual or group exposed to the risk factor is regularly exposed to another risk factor*.

The distinction between specific and non-specific diseases will be dealt with in the next section. This section directly addresses the question of whether epidemiological evidence can prove causation in an individual plaintiff, which is both a scientific and a legal question. A satisfactory approach must separate the scientific and legal parts of the question, and then proceed by applying scientific expertise to the scientific part, and legal expertise to the legal part [1,2].

The scientific question is difficult because it does not fall within the scope of any single science. Epidemiology is concerned with population health but not individuals, while medicine is concerned with individuals but not populations. There is no medical way to tell whether smoking caused a particular person’s lung cancer. Therefore, this question must be answered by logical inference from the epidemiological evidence.

The measure of relative risk (RR) tells us how many times more common a disease is among those exposed to a given risk factor than among those who are unexposed. A high RR is one piece of evidence that may be used to infer that exposure to a given risk factor causes the disease at an epidemiological level, within the population as a whole. The science of epidemiology is concerned with making such causal inferences at the population level. Merely calculating the RR and noting that it is high does not allow a solid causal inference to be drawn. However, in the case of smoking and lung cancer, there is no question that, at the population level, smoking causes lung cancer, and that the RR is high for all kinds of lung cancer (over 2, and often over 10, or higher). There is no question that the high RR of lung cancer among smokers compared to non-smokers is caused by smoking [3,4].

The next question is what this scientific fact about populations means for the individual members of those populations.

If we make certain assumptions, it is possible to use the RR to identify some facts relating to the probability that an individual’s lung cancer was caused by smoking. We cannot prove with certainty that an individual’s lung cancer was caused by smoking, but we can estimate a probability.

Suppose that smokers within a given population of interest are, on average, 20 times as likely to get lung cancer as non-smokers. Then, for a sample of 20 randomly selected smokers with lung cancer, we can infer that if none of them had smoked, on average one of them would have developed lung cancer. That is really just another way of affirming the scientific fact about the population that smoking causes lung cancer, with an RR of 20. Thus, in a given randomly selected group of 20 people with lung cancer, other things being equal, the chance that one of them would have developed lung cancer as a non-smoker is 1/20, or 0.05.

This simple calculation tells us how likely it is that a person would have developed lung cancer, if they had not smoked.

We want to know something slightly different: how likely it is that smoking caused lung cancer. The difference is that smoking can be a cause of lung cancer even for people who would have developed it anyway (a case of what philosophers call preemption [5,6]). Genetics could potentially have caused lung cancer in the absence of smoking, but if that person smokes, then either (a) smoking and genetics might together cause lung cancer, or (b) smoking alone might cause lung cancer.

This means that we cannot use RR to estimate the probability of causation (PC) itself directly [1,2,7-13]. However, we can use RR to estimate a lower bound on the PC, assuming that the RR > 1 [7]. This means that we may in some situations be able to prove that causation is at least probable enough to satisfy the purposes of a legal action [1,2].

In order to calculate the lower bound on the PC, we can use this PC inequality formula [1,2]:

$$PC\geq 1-\frac{1}{RR}$$

In our example, where the RR=20, the lower bound on the PC is 1-1 ⁄ 20=1 - 0.05=0.95, or 95%. This means that it is at least 95% probable that smoking caused this individual’s lung cancer, assuming that the individual we picked is a randomly selected smoker from a group to which this epidemiological evidence can reasonably be applied.

From the above analysis, we may draw five conclusions.

1) Epidemiological data can be used to estimate a lower bound on the PC, employing the formula set out and explained above, provided that no other factors indicate that causation is more or less likely in this particular case (e.g., that the individual plaintiff is not typical or belongs to a group to which the epidemiological evidence does not reasonably apply).
2) When the RR>2, the PC will be >50%, provided the conditions mentioned in (1) are satisfied.
3) The probability of smoking being the cause of the lung cancer in the cases of Gap and Eul, addressed in this decision, was over 50% if the relative risk was greater than 2, and was therefore was proven on the balance of probabilities by the epidemiological evidence provided that the RR > 2 and that the conditions in (1) were satisfied.
4) This calculation provides an estimate of a lower bound on the probability, provided that the conditions in (1) are satisfied, and does not outweigh or overrule other evidence, but must be weighed alongside it to determine the overall balance of probabilities in light of all the available evidence.
5) When RR<2 the PC may still be high (>50%) because the estimate only gives a lower bound. Thus, in this situation (RR<2), epidemiological evidence on its own does not say anything about whether causation is more likely than not, although it may be more informative when combined with other evidence.

## SPECIFIC AND NON-SPECIFIC DISEASES

The above-cited passage from the judgment (4[A]) distinguishes “specific” from “non-specific” diseases. However, the method presented above for estimating a lower bound on the PC makes no reference to this distinction. That is because the distinction between “specific” and “non-specific” diseases is not scientifically real.

The situation can be clarified as follows. First, it is true that some diseases are defined by reference to a certain cause, or to certain causes. Thus, a case of diarrhea and fever is not cholera unless *Vibrio cholerae* is present in the small intestine. This is a matter of definition [2,14-16]. Some diseases, such as swine fever, have more than one defining cause (swine fever requires the synergistic action of a bacterium and a virus [14]).

Second, it is also true that there are many diseases for which no defining causes are known, and perhaps none exist. The majority of cancers are like this, with a small number of exceptions, such as cervical cancer, which is exclusively or almost exclusively caused by the human papillomavirus [17].

When the Court refers to “specific diseases” and “non-specific diseases,” it appears to mean diseases for which a defining cause is known and not known, respectively. The Court appears to reason that, when a defining cause is known, we can confidently infer that the exposure of a patient to that defining cause must have been the cause of the ensuing disease; while if the disease is non-specific, we cannot tell whether a certain exposure, which is sometimes causal, was causal on this occasion.

This line of reasoning is mistaken, both in its confidence about “specific diseases” and in its lack of confidence about “non-specific diseases.”

Regarding the former, the term “specific disease” really ought to be “specific cause.” All diseases have many causes [18-21]. For example, among the causes of cholera is the consumption of water that has been in contact with human excrement. If a plaintiff develops fever and diarrhea after drinking such water, and dies, without ever receiving a positive diagnosis of cholera or a microscopic identification of *Vibrio cholerae* in the small intestine, that plaintiff is in the same position as a plaintiff in the case of a so-called “non-specific disease” such as lung cancer. Many things can cause fever and diarrhea, just as multiple causes of lung cancer are present. Therefore, no distinction between “specific” and “non-specific” diseases may be relied upon in the way that the Court hopes.

A more relevant distinction is that between two kinds of causes of disease: those that are both necessary and circumstantially sufficient for a disease (such as *Vibrio cholerae* in the small intestine, for cholera), and those that are not. By “specific disease,” the Court appears to mean a disease for which such a cause is known. However, even for a disease that has a known cause or causes meeting this criterion, we may be forced to infer whether the cause was operative in the circumstances, and thus whether what the plaintiff suffered really was a case of that disease—for example, whether this really was a case of cholera or not.

The term “non-specific diseases” should be re-interpreted as referring to diseases for which no cause that is both necessary and circumstantially sufficient is yet known. Regarding such diseases, the Court holds that as long as it is possible that an individual was exposed to another risk factor, it is possible that the disease was not caused by the risk factor that is the subject of the case; and therefore, it is impossible to prove causation.

This is correct only if proving causation requires proving with a probability of 100%, or certainty, that the risk factor in question was the cause. If, however, legal proof requires showing that the probability is above some threshold, for example 50%, then that is clearly a fallacious line of reasoning. It confuses proving that causation is certain (a probability of 100%) with proving that it is probable (a probability greater than some threshold, normally 50% in civil lawsuits).

I draw four conclusions from this analysis.

1) The distinction between “specific” and “non-specific” diseases does not correspond to a scientific distinction between different kinds of disease.
2) A distinction may be drawn between diseases for which defining causes are known and those for which they are either unknown or do not exist.
3) A distinction may be drawn between causes that are defining (i.e., necessary and circumstantially sufficient) for a given disease, and those that are not; every disease has non-defining causes, but only some have defining causes.
4) This distinction does not correspond to any distinction between diseases whose causes can be inferred from epidemiological evidence and those whose causes cannot. Difficulties of the same kind may arise in either case, and may be overcome under the conditions set out in the previous section.

## WARNINGS ABOUT THE DANGERS OF SMOKING

In addition to its findings on causation, the Court held that a defect in expression concerning the risks of smoking did not exist because these risks were widely known. Some of the reasons expressed in 1(B)(2) are as follows:

…*in 1962, the U.S. Surgeon General’s Report released study results showing that smoking is the main cause of lung cancer; these British and American studies were also reported in Korea by newspapers and other media. From then on to the 1990s, dozens of newspaper and media reports announced that cigarettes are hazardous to health and increase mortality rates by causing various diseases such as lung cancer, … due to the above media reports and legal regulations, the fact that smoking may cause various diseases, such as cancer, in the respiratory system, including the lungs, was widely recognized by cigarette smokers and society overall*…

Regardless of the narrow legal correctness of this finding, the profession of epidemiology has reason to be concerned about it for two reasons.

First, on factual grounds, it is far from clear that the dangers of smoking are widely known. In particular, at the age at which long-term smokers begin smoking, it is not clear that these dangers are fully understood. Often, long-term smokers begin smoking as children. Given the highly addictive and habit-forming nature of smoking, education at a point later in life may be less effective, since the freedom to choose is constrained if one has already begun smoking. Even if adults are aware of the dangers of smoking, the important question is whether young children are aware, or even capable of fully comprehending the dangers.

Second, supposing that it is granted that they are aware, then a serious question of legal policy arises. Awareness of the dangers of smoking did not come about easily. It has arisen from the strenuous efforts of a large number of epidemiologists, and many others involved in public health. These people have sought to raise awareness of the dangers of smoking, with the goal of reducing the number of deaths and illnesses caused by smoking.

It is a logical consequence of the Court’s reasoning that, had these efforts to raise awareness not been made, the tobacco companies would have been obliged to exert more effort to raise awareness of the dangers of smoking. Such efforts would have incurred additional costs and reduced the profitability of manufacturing cigarettes. These costs have in fact been born by professions (such as epidemiology) and institutions involved in public health. Is it right that this judgement has relieved the tobacco companies of contributing towards these costs, and allowed them to retain the portion of their profit arising from the largely altruistic efforts of the public health professions? This is a question of legal policy, and a moral question, which arises even if the decision is legally correct in a narrow sense.

## CONCLUSION

Contrary to the Court’s finding, epidemiological evidence can be used to estimate a lower bound on the PC in certain circumstances, as described here, provided that due care is taken to use the epidemiological evidence correctly. It is not determinative of the question of causation and must be considered alongside any other evidence relevant to causation. Also contrary to the Court’s finding, there is no distinction between “specific” and “non-specific” diseases that might prevent causation being established to a certain probability, as opposed to being proven with 100% certainty. Epidemiologists and others involved in public health might have cause for concern about the implications of the Court’s findings in relation to warnings about the dangers of tobacco, since these findings relieve tobacco companies of the costs of raising awareness about the dangers of tobacco, at the expense of the epidemiologists and others involved in public health.
